# Retraction: Evaluation of sleep quality and duration using wearable sensors in shift laborers of construction industry: a public health perspective

**DOI:** 10.3389/fpubh.2024.1521294

**Published:** 2024-11-05

**Authors:** Frontiers Editorial Office

The journal retracts the 20 September 2022 article cited above.

Following publication, concerns were raised regarding the ethical approval for this study. An investigation was conducted in accordance with Frontiers' policies, and it was confirmed that, contrary to the statement in the article, the study was not approved by Ethics Committee of the SRM Hospital and Research Centre. Lack of appropriate ethical approval is a breach of Frontiers' guidelines and those of the Committee on Publication Ethics. As such, this article is being retracted.

This retraction was approved by the Chief Executive Editor of Frontiers. The authors received a communication regarding the retraction and had a chance to respond. This communication is recorded by the publisher.

